# Effects of immune cells in mediating the relationship between gut microbiota and myelodysplastic syndrome: a bidirectional two-sample, two-step Mendelian randomization study

**DOI:** 10.1007/s12672-024-01061-6

**Published:** 2024-05-31

**Authors:** Zuxi Feng, Minjing Liao, Xuege Guo, Lijuan Li, Liansheng Zhang

**Affiliations:** 1 Department of Hematology, Lanzhou University Second Hospital, Lanzhou, 730000 China; 2 Second Clinical Medical College, Lanzhou University, Lanzhou, China

**Keywords:** Myelodysplastic syndrome, Gut microbiota, Mendelian randomization, Immunophenotypes

## Abstract

**Background:**

The definitive establishment of a causal relationship between gut microbiota and myelodysplastic syndrome (MDS) has not been achieved. Furthermore, the involvement of immune cells in mediating the connection between gut microbiota and MDS is presently unclear.

**Methods:**

To elucidate the bidirectional correlation between gut microbiota and MDS, as well as to investigate the mediating role of immune cells, a bidirectional two-sample, two-step Mendelian randomization (MR) study was conducted. Summary statistics were obtained from genome-wide association studies (GWAS), including MDS (456,348 individuals), gut microbiota (18,340 individuals), and 731 immune cells signatures (3757 individuals).

**Results:**

Genetically predicted eight gut microbiota traits were significantly associated with MDS risk, but not vice versa. Through biological annotation of host-microbiome shared genes, we found that immune regulation may mediate the impact of gut microbiota on MDS. Subsequently, twenty-three immunophenotypes that exhibited significant associations with MDS risk and five of these immunophenotypes were under the causal influence of gut microbiota. Importantly, the causal effects of gut microbiota on MDS were significantly mediated by five immunophenotypes, including *CD4* +*T cell %leukocyte*, *CD127 on CD45RA − CD4 not regulatory T cell*, *CD45 on CD33* + *HLA DR* + *WHR*, *CD33 on basophil*, and *Monocyte AC*.

**Conclusions:**

Gut microbiota was causally associated with MDS risk, and five specific immunophenotypes served as potential causal mediators of the effect of gut microbiota on MDS. Understanding the causality among gut microbiota, immune cells and MDS is critical in identifying potential targets for diagnosis and treatment.

**Supplementary Information:**

The online version contains supplementary material available at 10.1007/s12672-024-01061-6.

## Introduction

Myelodysplastic syndrome (MDS) is a complex hematologic disorder marked by impaired cell maturation in the bone marrow, resulting in clinical manifestations such as anemia, bleeding, and infections. MDS carries significant clinical implications, predominantly stemming from cytopenias, which can result in various morbidities [1]. The Revised International Prognostic Scoring System currently serves as the most extensively utilized prognostic scoring system to customize treatment for patients diagnosed with MDS [2]. The evaluation of individual risk aids in identifying appropriate candidates with an adverse prognosis, eligible for aggressive initial interventions, notably allogeneic stem cell transplantation [3]. Consequently, it is imperative to undertake further investigation into additional risk factors associated with the diagnosis and treatment of MDS.

Human body harbors an intricate ecosystem of microorganisms, encompassing bacteria, viruses, fungi, and other life forms, which collectively constitute the microbiome. While various bodily organs host unique microbial communities, considerable focus in biomedical research has centered on the complex microbial assembly within the gastrointestinal tract [4]. In the last decade, substantial advancements have been achieved in comprehending the composition, functions, and impacts of gut-dwelling organisms on human health and pathology [5]. The mutual interplay between the microbiome and the intestinal immune system substantially contributes to the maintenance of mucosal homeostasis [6]. Lately, growing evidence underscores the significant influence of the microbiome on the effectiveness of tumor immunotherapy, with a particular focus on immune checkpoint inhibitors [7]. The intricate interplay between the gut microbiota and the host’s immune system plays a pivotal role in preventing infections and maintaining immune equilibrium. The increasingly rich foundational research concerning the association between gut microbiota and hematologic malignancies provides a strong basis for potential therapeutic interventions aimed at modulating the microbiome in these conditions [8]. In the past decade, comprehensive study has demonstrated the substantive involvement of the gut microbiota, particularly gastrointestinal commensal bacteria, in the development of hematological malignancies [9]. The definitive establishment of a causal relationship between gut microbiota and MDS has not been achieved. Furthermore, the involvement of immune cells in mediating the connection between gut microbiota and MDS is presently unclear.

Because it is challenging to obtain real-time microbiome data in clinical settings, there is a lack of research elucidating the causal relationship between specific microbial changes and MDS. Furthermore, the intricate influence of extraneous variables such as lifestyle, environment, host gene mutations, and potential reverse causality presents a formidable challenge in disentangling the interplay between the host-microbiome genome. To address these complexities, the adoption of two-sample Mendelian Randomization (MR) emerges as a robust methodology. This approach employs hereditary genetic variations as instrumental variables (IVs) to discern causal relationships between exposure and outcome [10]. Presently, MR is extensively utilized to investigate the causal relationships between gut microbiota and various diseases [11]. Presently, no scholars have utilized the MR method to investigate matters concerning MDS.

Thus, we propose a causal association between gut microbiota and MDS, with its effects partly mediated through immune cells. We performed MR analyses to explore the causal links between gut microbiota and MDS, and further investigate the functions of 731 immunophenotypes in mediating the connections between gut microbiota and MDS. Enhancing our comprehension of the causal relationships among gut microbiota, immune cells, and MDS risk holds promise for identifying novel targets for prevention and treatment of MDS in patients.

## Materials and methods

### Study design

**Fig. 1:**
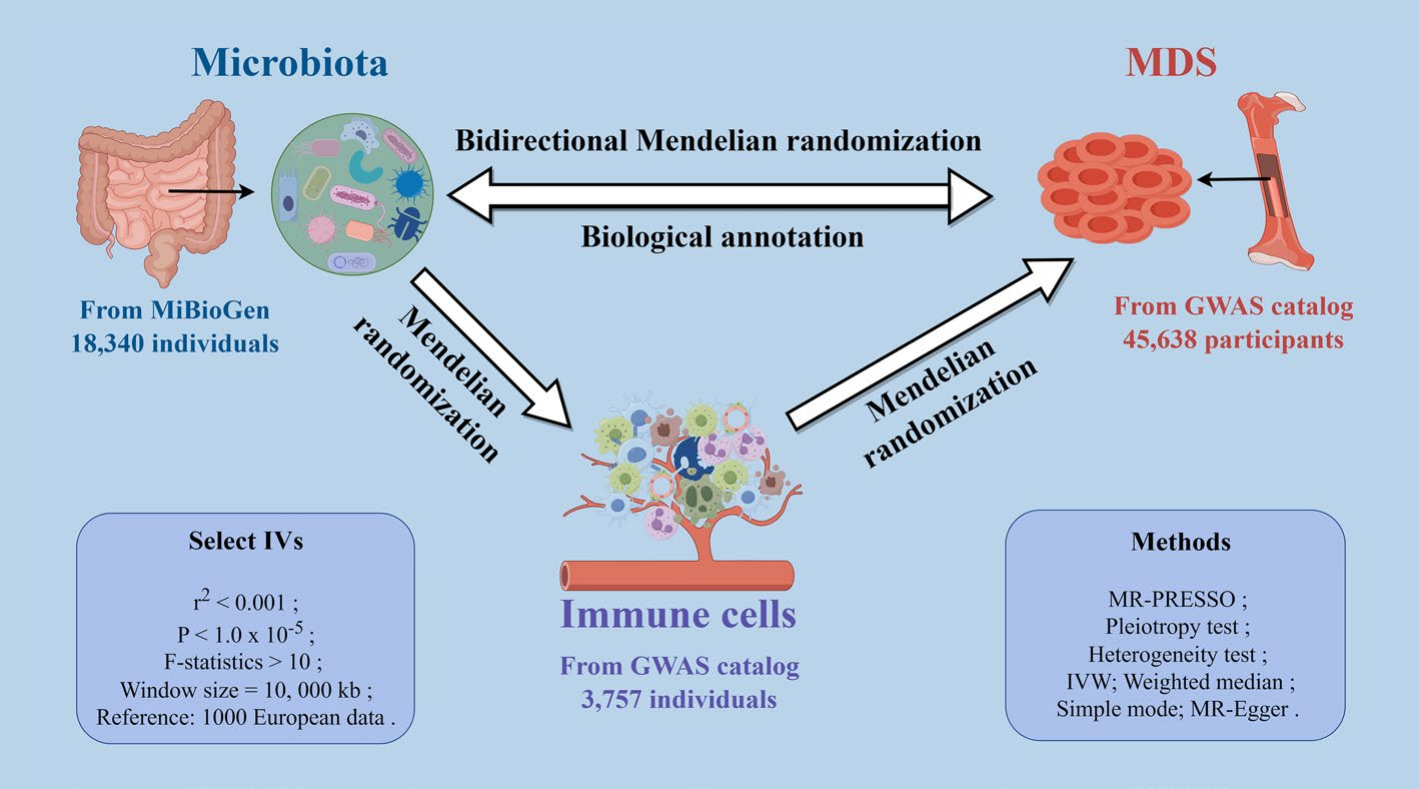
The study design for the mediation of relationship between gut microbiota and myelodysplastic syndrome by immune cell traits. *MDS*, myelodysplastic syndrome, *IVs* instrumental variables, *GWAS* genome-wide association studies, *MR-PRESSO* MR pleiotropy residual sum and outlier, *IVW* inverse-variance weighted

The comprehensive research design outlined in Fig. 1. This study employed a bidirectional two-step, two-sample MR approach, utilizing publicly available datasets to investigate the potential mediating roles of 731 immune cell traits between gut microbiota and MDS risk. Initially, we estimated the causal effect of gut microbiota on MDS. Subsequently, we conducted two-step MR analyses to ascertain the mediation effects of 731 immune cell traits. Firstly, we examined the causal effects of the 731 immune cell traits on MDS. Next, we evaluated the causal effects of gut microbiota the identified potential mediators. These evaluations were then multiplied to derive the mediation effects of the potential mediator on the outcome of MDS.

### Data sources

In this research, we extracted summary statistics related to MDS from a previously published GWAS available at http://ftp.ebi.ac.uk/pub/databases/gwas/summary_statistics/GCST90042001-GCST90043000/GCST90042662/. This GWAS incorporated data from the UK Biobank and encompassed 456,348 individuals with European ancestry [12]. Additionally, summary statistics concerning the human gut microbiota in our investigation were derived from a GWAS involving 18,340 participants (https://mibiogen.gcc.rug.nl) [13]. The GWAS Catalog provides publicly available summary statistics for 731 immune cells (with accession numbers ranging from GCST90001391 to GCST90002121) [14]. A comprehensive analysis was conducted on a total of 731 immunophenotypes, involving various types of data, such as absolute cell counts (AC), median fluorescence intensities (MFI) which represented surface antigen levels, morphological parameters (MP), and relative cell counts (RC). The initial GWAS on immune traits utilized data from 3757 individuals of European descent, with no identified overlap in cohorts [15].

### IVs

Initially, we excluded 15 unidentifiable microbial attributes, leaving us with 196 distinct microbial features. Subsequently, we selected IV employing a less stringent significance threshold of *p* < 5.0 × 10^–6^ (Table S1). To prevent biased results stemming from linkage disequilibrium (LD), we performed clumping analysis on European samples from the 1000 Genomes project using the TwoSampleMR package, with stringent criteria (r^2^ < 0.001 and a 10,000 kb window size). With an r^2^ value of less than 0.001, we retained only the single nucleotide polymorphism (SNP) possessing the lowest p-value. To gauge the strength of the IVs, we computed F-statistics using the provided equation R^2^ = 2 × MAF × (1 − MAF) × β2, F = R^2^ × (N − 1 − k)/(1 − R^2^), where R^2^ represents the variance explained by the IVs, “N” denotes the data sample size, and “k” represents the count of SNPs included in the instrument [16]. In the backward MR analysis, we chose IVs linked to MDS at cutoff levels 5.0 × 10^–6^ (Table S2), we adopted 5.0 × 10^–6^ for the selection of IVs due to the limited availability of SNPs associated with MDS at the GWAS significant level, in accordance with previous studies. Besides, according to recent research [14], the significance threshold for IVs associated with each immune trait was established at 1.0 × 10^–5^ (Table S3a). IVs must not influence the outcome through pathways other than the exposure of interest. To escape this violation, we screened for common SNPs associated with MDS traits using the LDtrait Tool (https://ldlink.nih.gov/), the results are displayed in Table S3b.

### Biological annotation

Initially, we annotated genes associated with IVs' positions between gut microbiota and MDS using the GWAS catalog provided with FUMA [17]. To construct protein–protein interaction (PPI) networks, we employed STRING (https://string-db.org/) and visualized network using Cytoscape (version 3.8.2). Employing the Maximal Clique Centrality (MCC) approach, we identified the top 10 hub genes, emphasizing genes crucial for maintaining network stability due to their numerous interconnecting links [18]. To evaluate the impact of these hub genes comprehensively, we conducted phenome-wide association studies (PheWAS), exploring their pleiotropic effects across 28 different domains using the GWAS ATLAS and summary data for 4756 complex traits and illnesses [19].

### Statistical analysis

This study employed various methodologies, including MR-PRESSO, IVW, weighted median, MR-Egger regression, simple mode, and weighted model [20]. Our primary MR analysis relied on the IVW method [21]. We assessed horizontal pleiotropy by testing genetic variant heterogeneity and confirming MR assumptions through the MR-Egger intercept [16]. We performed sensitivity analyses to account for the issue of horizontal pleiotropy. Moreover, we employed the MR-PRESSO approach for global and outlier tests to pinpoint potential anomalies. Following the identification of these potential outliers, we derived refined association outcomes by excluding them from the analysis [22]. The mediation effects were computed using the formula: β1 * β2. Here, β1 represents the impact of gut microbiota on immune cell traits, while β2 indicates the influence of mediators on MDS. Standard errors and confidence intervals (CIs) were determined using the delta method [23]. “Mendelian Randomization,” “TwoSampleMR,” and “MRPRESSO” packages in the open source statistical software R (version 4.2.3) were employed for MR analyses. The connections between the gut microbiota and MDS risk were quantified using odds ratio (OR) accompanied by 95% confidence interval (CI). The false discovery rate (FDR) method was used for multiple testing correction. Statistical significance was confirmed when p-values were below 0.05.

## Results

### Causal relationship between gut microbial and MDS

**Fig. 2:**
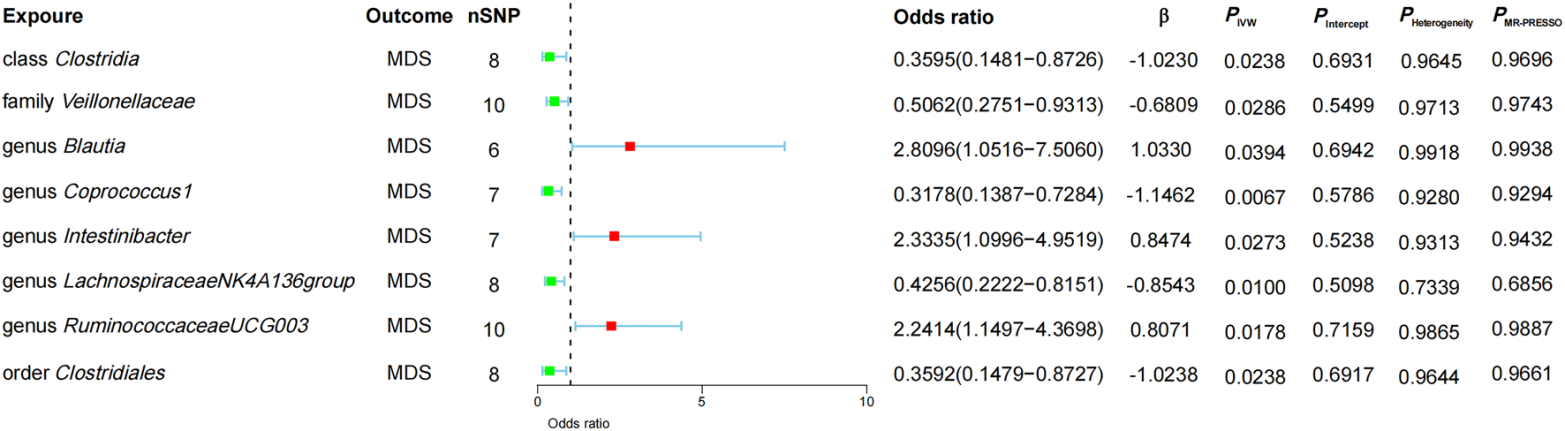
Mendelian randomization estimates using the IVW method to evaluate the causal impact of the gut microbiota on myelodysplastic syndrome. *MDS* myelodysplastic syndrome, *nSNP* number of single nucleotide polymorphisms, *P*_*IVW*_ p-value for Inverse-Variance Weighted, *P*_Intercept_ p-value for MR-Egger intercept, *P*_Heterogeneity_ p-value for Heterogeneity test, *P*_MR-PRESSO_ p-value for MR-PRESSO global test

In the forward MR analyses, our findings revealed positive relationships between MDS risk and the following microbiota features: genus *Blautia* (OR, 2.8096; 95%CI 1.0516 − 7.5060; P = 0.0394), genus *Intestinibacter* (2.3335; 1.0996 − 4.9519; 0.0273), and genus *RuminococcaceaeUCG003* (2.2414; 1.1497 − 4.3698; 0.0178) (Fig. 2). On the contrary, we found negative associations between MDS risk and the following microbiota features: class *Clostridia* (0.3595; 0.1481 − 0.8726; 0.0238), family *Veillonellaceae* (0.5062; 0.2751 − 0.9313; 0.0286), genus *Coprococcus1* (0.3178; 0.1387 − 0.7284; 0.0067), genus *LachnospiraceaeNK4A136group* (0.4256; 0.2222 − 0.8151; 0.0100), and order *Clostridiales* (0.3592; 0.1479 − 0.8727; 0.0238) (Fig. 2). Furthermore, sensitivity analyses including MR-Egger intercept, Heterogeneity test, and MR-PRESSO global test indicated no evidence of directional pleiotropy. The detailed results of MR analysis were outlined in Table S4. In the backward MR analyses, no substantial evidence was found to support the causal effects of MDS on eight microbial characteristics identified in the forward MR analyses. The detailed results of backward MR analysis were presented in Table S5.

### Biological annotation

**Fig. 3:**
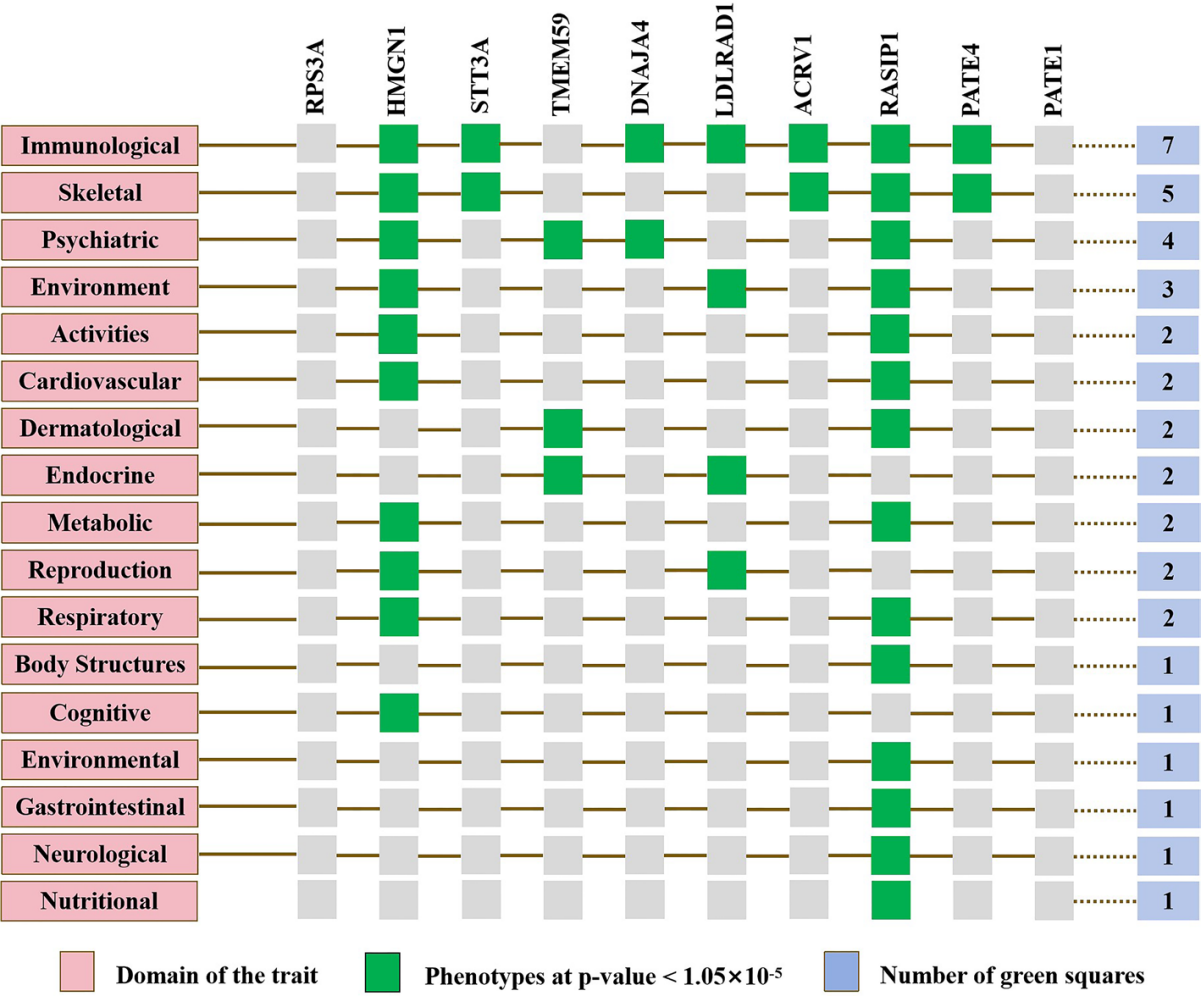
Biological annotation of 67 mapped genes between myelodysplastic syndrome and gut microbiota. PheWAS analyses with GWAS ATLAS. Traits ordered by domain, overall number of GWASs considered for these analyses: 4,756. Default p-value cutoff at 0.05 and Bonferroni corrected p-value = 1.05 × 10^–5^

To elucidate the biological mechanisms that connect gut microbiota to MDS, we annotated the IVs mapping onto 67 host-microbiome shared genes (Table S6). A tightly connected network of 67 shared proteins was identified through PPI network analysis (Table S7). To gain further insights, we used the MCC method to rank the top 10 nodes as hub genes in the PPI network (Table S8). To ascertain the potential influence of these hub genes on various traits, we conducted gene-set functional analysis. Gene-based PheWAS indicated that seven of the ten hub genes displayed heightened genetic signals associated with immunological domains (Fig. 3 and Table S9). This implies that immune regulation could be a potential mechanism through which gut microbiota influences MDS. Therefore, we speculate that immune cells serve as mediators between the microbiota and MDS.

### Effects of immune cells on MDS risk

**Fig. 4:**
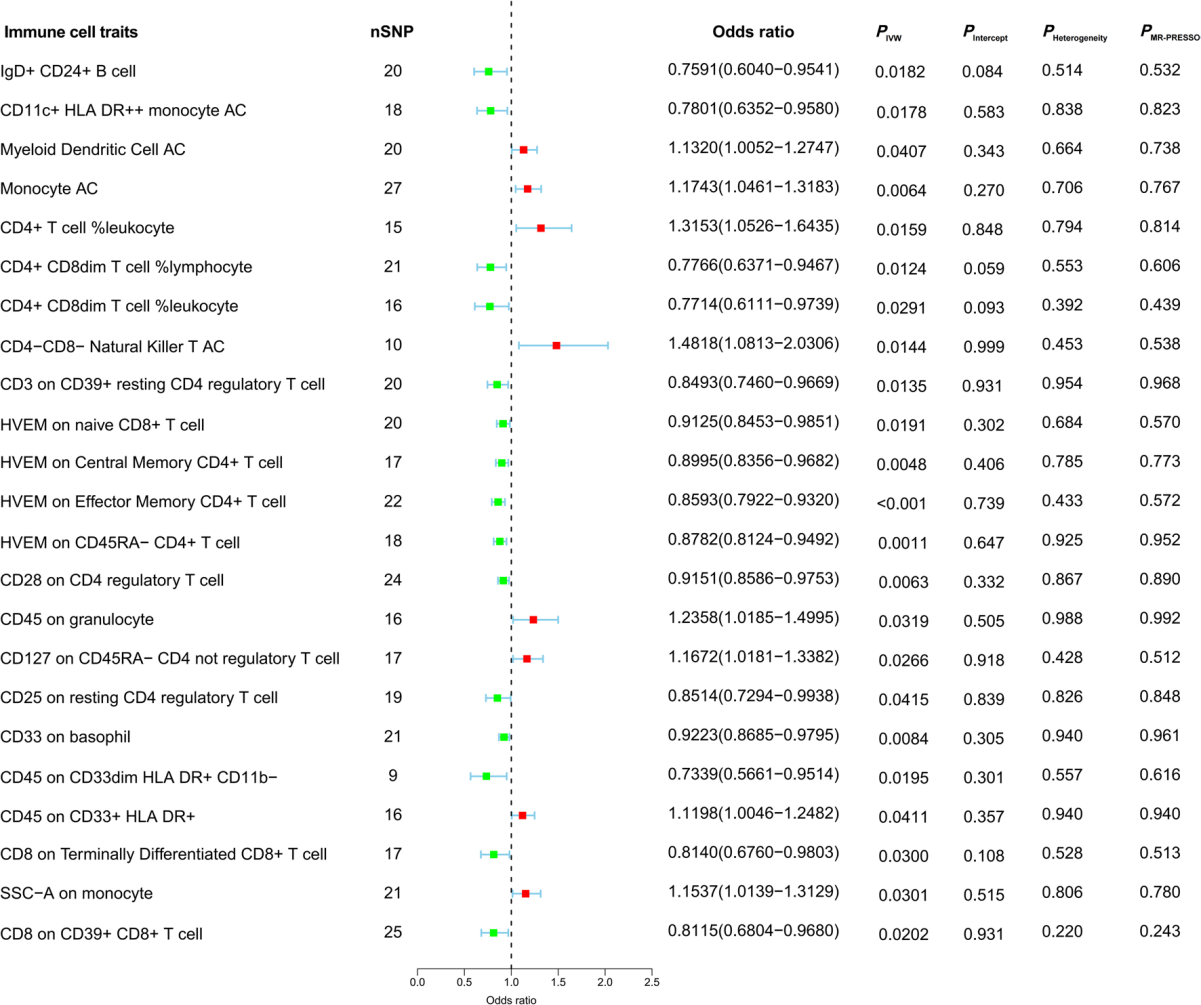
Mendelian randomization estimates were used to assess the causal effect of immune cell traits on myelodysplastic syndrome. *MDS* myelodysplastic syndrome, *nSNP* number of single nucleotide polymorphisms, *P*_*IVW*_ p-value for Inverse-Variance Weighted, *P*_Intercept_ p-value for MR-Egger intercept, *P*_Heterogeneity_ p-value for Heterogeneity test, *P*_MR-PRESSO_ p-value for MR-PRESSO global test

We detected protective effects of eight immunophenotypes on MDS: *Myeloid Dendritic Cell AC* (OR, 1.132; *P* = 0.0407), *Monocyte AC* (OR, 1.1743; *P* = 0.0064), *CD4* +*T cell %leukocyte* (OR, 1.3153; *P* = 0.0159), *CD4-CD8- Natural Killer T AC* (OR, 1.4818; *P* = 0.0144), *CD45 on granulocyte* (OR, 1.2358; P = 0.0319), *CD127 on CD45RA- CD4 not regulatory T cell* (OR, 1.1672; *P* = 0.0266), *CD45 on CD33* + *HLA DR* + (OR, 0.0411; *P* = 0.0394), and *SSC-A on monocyte* (OR, 1.1537; *P* = 0.0301) (Fig. 4). On the contrary, harmful effects of fifteen immunophenotypes on MDS: *IgD* + *CD24* + *B cell* (OR, 0.7591; *P* = 0.0182), *CD11c* + *HLA DR* +  + *monocyte AC* (OR, 0.7801; *P* = 0.0178), *CD4* + *CD8dim T cell %lymphocyte* (OR, 0.7766; *P* = 0.0124), *CD4* + *CD8dim T cell %leukocyte* (OR, 0.7714; *P* = 0.0291), *CD3 on CD39* +*resting CD4 regulatory T cell* (OR, 0.8493; *P* = 0.0135), *HVEM on naive CD8* + *T cell* (OR, 0.9125; *P* = 0.0191), *HVEM on Central Memory CD4* +*T cell* (OR, 0.8995; *P* = 0.0048), *HVEM on Effector Memory CD4* +*T cell* (OR, 0.8593; *P* = 0.0003), *HVEM on CD45RA- CD4* +*T cell* (OR, 0.8782; *P* = 0.0011), *CD28 on CD4 regulatory T cell* (OR, 0.9151; *P* = 0.0063), *CD25 on resting CD4 regulatory T cell* (OR, 0.8514; *P* = 0.0415), *CD33 on basophil* (OR, 0.9223; *P* = 0.0084), *CD45 on CD33dim HLA DR* + *CD11b-* (OR, 0.7339; *P* = 0.0195), *CD8 on Terminally Differentiated CD8* +*T cell* (OR, 0.814; *P* = 0.03), and *CD8 on CD39* + *CD8* +*T cell* (OR, 0.8115; *P* = 0.0202) (Fig. 4). Besides, sensitivity analyses including MR-Egger intercept, Heterogeneity test, and MR-PRESSO global test indicated no evidence of directional pleiotropy. The comprehensive results of MR analysis were displayed in Table S10. In an effort to address potential pleiotropy, we scanned all SNPs used as IVs in our study using the LDtrait tool (Table S3b). This led to the identification of 23 common SNPs associated with pleiotropic traits, and relevant literature was reviewed to explore their connections to MDS. We conducted MR analyses using the IVW method after excluding these pleiotropic SNPs (Table S3c). The results indicate that after individually removing five SNPs (rs511713, rs10858526, rs35290870, rs111693583, and rs524655), the p-value in the IVW method remains greater than 0.05 (Table S3c). Furthermore, we carefully examined the correlation between the pleiotropic traits associated with these five SNPs and MDS. There are no previous research findings proving the correlation between the pleiotropic traits associated with rs511713, rs10858526, rs35290870, and rs524655 and MDS. However, rs111693583 is associated with dozens of pleiotropic traits, some of which have been proven to have a causal relationship with MDS. Therefore, rs111693583 is an important pleiotropic variant, and the causal relationship between the CD4 + CD8dim T cell %leukocyte-MDS trait associated with rs111693583 is unreliable and requires further in-depth analysis.

### Effects of gut microbial on immune cells.

**Fig. 5:**
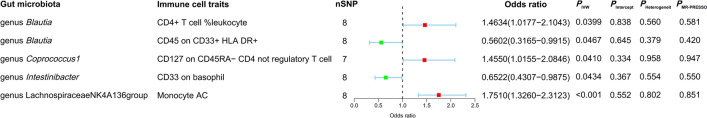
Mendelian randomization estimates were obtained to assess the causal influence of gut microbiota on immune cell traits. *nSNP* number of single nucleotide polymorphisms, *P*_*IVW*_ p-value for Inverse-Variance Weighted, *P*_Intercept_ p-value for MR-Egger intercept, *P*_Heterogeneity_ p-value for Heterogeneity test, *P*_MR-PRESSO_ p-value for MR-PRESSO global test

According to the above results, twenty-three immunophenotypes were identified as potential mediators. Subsequently, a thorough MR analysis was undertaken to investigate the impact of gut microbiota on these mediators. We found that genus *Blautia* could increase the level of *CD4* + *T cell %leukocyte* (OR, 1.4634; *P* = 0.0399) and decrease the level of *CD45 on CD33* + *HLA DR* + (OR, 0.5602; *P* = 0.0467). Besides, genus *Coprococcus1* increase the level of *CD127 on CD45RA- CD4 not regulatory T cell* (OR, 1.455; *P* = 0.041), genus *Intestinibacter* decrease the level of *CD33 on basophil* (OR, 0.6522; *P* = 0.0434), genus *LachnospiraceaeNK4A136group* increase the level of Monocyte AC (OR, 1.751; *P* < 0.001) (Fig. 5). Sensitivity analyses including MR-Egger intercept, Heterogeneity test, and MR-PRESSO global test indicated no evidence of directional pleiotropy. The detailed results of MR analysis were presented in Table S11.

### Mediation effects of immune cells on MDS.

In light of the outcomes from the MR analyses, five immunophenotypes were identified as potential mediators, and their respective mediation effects, along with the 95% CI, were calculated. The mediation effects and the 95% CI are presented in Table 1. In the correlation between genus *Blautia* and MDS, two specific mediators were distinguished, including *CD4* +*T cell %leukocyte* (mediation effect: 0.104; 95% CI − 0.047,0.256) and *CD45 on CD33* + *HLA DR* + *WHR* (mediation effect: − 0.066; 95% CI − 0.397, 0.265). In the relationships of genus *Coprococcus1* with MDS, *CD127 on CD45RA − CD4 not regulatory T cell* (mediation effect: 0.058; 95% CI − 0.079, 0.194) was found to be the mediator. *CD33 on basophil* (mediation effect: − 0.035; 95% CI − 0.212, 0.143) was considered to be mediators for the impact of genus *Intestinibacter* on MDS. *Monocyte AC* (mediation effect: 0.090; 95% CI − 0.067, 0.247) was also found to be the mediator between genus *LachnospiraceaeNK4A136group* and MDS. The detailed results of mediation effects were presented in Table S12.

**Table 1:** The mediating effects of mediators in the causal association linking gut microbiota to MDS

Mediator	The effect of exposure on outcome β (95% CI)	The effect of exposure on mediator β1 (95% CI)	The effect of mediator on outcome β2 (95% CI)	Mediation effect (95% CI)
CD4 + T cell %leukocyte	1.033 (0.050, 2.016)	0.381 (0.018, 0.744)	0.274 (0.051, 0.497)	0.104 (− 0.047, 0.256)
CD45 on CD33 + HLA DR +	1.033 (0.050, 2.016)	− 0.579 (− 1.150, − 0.009)	0.113 (0.005, 0.222)	− 0.066 (− 0.397, 0.265)
CD33 on basophil	− 1.146 (− 1.976, − 0.317)	− 0.427 (− 0.842, − 0.013)	− 0.081 (− 0.141, − 0.021)	− 0.035 (− 0.212, 0.143)
Monocyte AC	0.847 (0.095, 1.600)	0.560 (0.282, 0.838)	0.161 (0.045, 0.276)	0.090 (− 0.067, 0.247)
CD127 on CD45RA − CD4 not regulatory T cell	− 0.854 (− 1.504, − 0.204)	0.375 (0.015, 0.735)	0.155 (0.018, 0.291)	0.058 (− 0.079, 0.194)

### Sensitivity analyses

Several sensitivity analyses were executed to assess the robustness of our findings. First, the F-statistic of IVs used in all MR analysis were calculated (Tables S9–S12). We then conducted a heterogeneity test to obtain the heterogeneity in our estimates. Besides, for the identification of potential pleiotropic effects, we utilized the p-value linked to the MR-Egger intercept. To evaluate the effects of genetically predicted gut microbiota on MDS outcomes, we employed various methods, including weighted mode, simple mode, MR-Egger, and weighted median. The MR-PRESSO global test was implemented to address heterogeneities and identify and remove outliers in our analysis, thereby correcting horizontal pleiotropy. Subsequent to the removal of outliers, the results remained consistent with the original findings across all positive outcomes. The consistency observed in the sensitivity analyses bolsters the robustness of the causal inferences drawn from the primary analyses. Tables S4–S5 and Tables S10–S11 present the results of our sensitivity analyses. In essence, these findings collectively affirm the strength and dependability of our primary conclusions that the genetic predisposition to gut microbiota is intricately associated with heightened susceptibility to MDS. Moreover, our research highlights the potential involvement of five distinct immune cell traits in facilitating the causal relationship between gut microbiota and MDS.

## Discussion

Individuals suffering from MDS often exhibit debilitating constitutional symptoms, including persistent fatigue, recurrent fever, and heightened vulnerability to severe and unusual infections [24]. Numerous studies have demonstrated the significant influence of lifestyle and environmental factors in the development of MDS. However, genetics also accounts for a substantial proportion of MDS cases [25, 26]. The swift progress in macro-genomics and diverse sequencing technologies, including 16S rRNA gene amplicon sequencing and shotgun metagenome sequencing, has facilitated the analysis of variations in the human gut microflora by researchers [27]. Recent extensive investigations have emphasized the crucial role of the gut microbiota in oncology, encompassing hematological malignancies [28–30]. However, the causal relationship between gut microbiota and MDS remains unknown. Thus, our investigation employed a two-sample Mendelian randomization analysis coupled with biological annotation, harnessing the established computational framework and statistical robustness of microbiome GWAS. We explored potential causal associations between microbiota features and MDS. Besides, we identified potential causal mediators which could potentially mediate the interaction between gut microbiota and MDS.

The configuration of the gut microbiome is intricately linked to the modulation of hematopoiesis, gastrointestinal health, and immune response. Dunn et al. observed a reduction in stool diversity, a reduction in butyrate-producing bacteria such as *Blautia*, and an elevation in opportunistic pathogens as a consequence of antibiotic and antifungal treatments. These discoveries hold implications for the management of leukemia and lymphoma [31]. Here, we found that genera (*Blautia*, *Intestinibacter* and *RuminococcaceaeUCG003*) were risk factors for MDS. Conversely, class *Clostridia*, family *Veillonellaceae*, genera (*Coprococcus1*, *LachnospiraceaeNK4A136group*), and order *Clostridiales* were protective factors. Existing research points to a noteworthy association between the broader equilibrium of gut microbial communities and immune responses [32]. A study demonstrated that the utilization of the genetically modified probiotic EcN-Sj16 resulted in heightened levels of beneficial *Ruminococcaceae*, elevated butyrate production, and the modulation of immunoregulatory Treg cells [33]. The class *Clostridia* represents a prevalent assemblage of gut bacteria within the Gram-positive bacterial group. Recent studies indicate a direct correlation between specific variations of *Clostridia* and human well-being, with select species potentially influencing both the human immune system and metabolism [34, 35]. In addition, family *Veillonellaceae* [36], genus *Lachnospiraceae* [37], and order *Clostridiales* [38] have also been shown to be associated with human tumor immunity. However, the role of these microbial features in blood diseases remains unclear. Biological annotation analyses identified 67 shared genes between gut microbiota and MDS. We identified the top 10 genes linking MDS with gut microbiota, and PheWAS analysis showed a correlation between these genes and immunological characteristics. Several studies have indicated that individuals with MDS exhibit immune irregularities, manifested by disrupted expression of various immune regulatory factors and disturbances in inflammatory signaling pathways [39, 40]. Thus, a potential mechanism linking gut microbiota and MDS may involve the modulation of immune system alterations through host-microbiome shared genes.

The implications of the gut microbiome on host immunity offer promising prospects for cancer prognosis and therapy. In the context of multiple myeloma, the immune system plays a central role in facilitating the interplay between gut microbiota and the chronic antigenic stimulation of B cells [41]. The immune-modulated gut microbiota might create an immunosuppressive environment, facilitating the progression and relapse of chronic myeloid leukemia and acute lymphoblastic leukemia [42]. A study reveals a plausible association between the gut microbiota and immune function in individuals diagnosed with diffuse large B-cell lymphoma, modulating the gut microbiota might improve immune response and treatment outcomes among patients affected by this condition [43]. The interplay between gut microbiota and the immune system may significantly impact the genesis, progression, and treatment outcomes of hematological malignancies [44]. Our large scale MR study found that twenty-three distinct immunophenotypes significantly associated with MDS risk, among which five were shown to be causally affected by gut microbiota. Therefore, we speculate that these five specific immunophenotypes, including *CD4* +*T cell %leukocyte*, *CD127 on CD45RA − CD4 not regulatory T cell*, *CD45 on CD33* + *HLA DR* + *WHR*, *CD33 on basophil*, and *Monocyte AC*, may mediate the interaction between the gut microbiota and MDS. CD4 +T-cells in MDS exhibit dysregulation, correlating with autoimmune hematopoietic suppression and impacting the response to immunosuppressive therapy, particularly in younger patients [45]. Besides, the role of CD33 on basophils in the transition from MDS to basophilic leukemia is noteworthy [46]. We then conducted a mediation analysis and identified that the impact of gut microbiota on the risk of MDS was partially mediated by five specific immunophenotypes. Considering the mechanistic perspective, the involvement of these immunophenotypes might significantly contribute to the link between the gut and MDS. The pivotal role of immunoregulation in the initiation and progression of MDS is widely recognized [47]. Although our findings indicate that immune phenotypes mediate the impact of gut microbiota on MDS, additional research is warranted to probe the specific molecular mechanisms underlying the gut microbiota-immunophenotypes-MDS axis.

Our study has several strengths and limitations. In this investigation, we employed a bidirectional MR approach for the first time to examine the causal relationships involving immune cells as mediators in the association between gut microbiota and MDS. Employing a bidirectional two-sample MR analysis allowed us to mitigate the biases stemming from reverse causation and confounders, which are primary limitations of conventional observational studies. Moreover, the utilization of summary data from GWAS in a two-sample MR framework not only reduced potential statistical limitations but also enhanced the statistical power, particularly in evaluating impacts on binary disease outcomes [48]. Furthermore, each SNP exhibited an F-statistic exceeding 10, ensuring robust statistical power. Additionally, a series of sensitivity analyses were performed to validate the consistent nature of the causal relationships. Despite these valuable insights, it is crucial to recognize some constraints inherent to the current study. We chose IVs linked to gut microbiota and immunophenotypes based on a threshold of *p* < 5.0 × 10^−6^, exceeding the standard threshold for genome-wide significance (*p* < 5 × 10^–8^). This higher threshold was necessary to ensure we had a sufficient number of IVs for our MR analyses. Furthermore, due to the limited sample size, the p-values obtained from the IVW method in this study did not yield significant results after FDR correction. In smaller sample sizes, estimates of FDR may become unstable, potentially leading to biased and inaccurate results. In the future, we expect to achieve more reliable results with MR through a significant increase in sample size, while also adhering to stricter criteria. Owing to the unavailability of individual level data, we were precluded from investigating whether additional potential variables in the subpopulation serve as mediators in the causal association between gut microbiota and MDS. Due to the limited availability of GWAS samples from diverse populations, the data used for MR in this study primarily originated from European populations (Table S13). This may potentially restrict the reliability of extrapolating the study findings to non-European populations. While our MR analyses have identified several potential causal connections, it is essential to underscore that these findings require further confirmation. The analysis methods employed in this study are limited, particularly in identifying potential causal variants. Therefore, it is advisable to incorporate a statistical fine-mapping method, such as SuSIE, to identify the potential causal variants from GWAS for the following MR study [49]. In this study, multiple common SNPs influence MDS by affecting different immune cell subtypes, and these SNPs have various pleiotropic traits [50]. Although we conducted thorough checks using the LDtrait tool and performed MR analysis after excluding SNPs, the presence of pleiotropic variants still makes the results less accurate. In our pursuit of a more comprehensive understanding, we must not only consider expanding the sample size but also focus on developing new techniques to enhance the statistical power of GWAS. Moreover, it is crucial to undertake further investigations into the underlying biological mechanisms, specifically concentrating on elucidating the specific molecular mechanisms of the gut microbiota-immunophenotypes-MDS axis.

## Conclusions

In summary, this large scale MR investigation provides robust support for the causal influence of gut microbiota on MDS risk. This relationship is partially mediated by five specific immunophenotypes. Understanding the causal connections among gut microbiota, immunophenotypes and MDS is vital in elucidating the pathogenesis of MDS and pinpointing potential targets for early intervention.

### Supplementary Information

Supplementary Material 1.

## Data Availability

Data used in this study are publicly available at http://ftp.ebi.ac.uk/pub/databases/gwas/summary_; https://mibiogen.gcc.rug.nl; https://string-db.org/;

## Code availability

Code used in this study are available at correspondence email.
